# Effect of Suturing and Adhesive Fixation on Free Gingival Graft Stability: An Ex‐Vivo Porcine‐Model Study

**DOI:** 10.1002/cre2.70345

**Published:** 2026-04-26

**Authors:** Kevimy Agossa, Khushboo Kalani, Parham Hazrati, Chi‐Fan Chen, Chen‐Lin Tsai, Dumitru Chele, Abdusalam E. Alrmali, Hom‐Lay Wang

**Affiliations:** ^1^ Department of Periodontics and Oral Medicine University of Michigan School of Dentistry Ann Arbor MI USA; ^2^ Univ. Lille, CHU Lille, U1008 ‐ Advanced Drug Delivery Systems Department of Periodontology School of Dentistry Lille France; ^3^ Oro‐maxillo‐facial Surgery and Oral Implantology Department State University of Medicine and Pharmacy “Nicolae Testemitanu” Chisinau Republic of Moldova; ^4^ Department of Oral Pathology, Oral Medicine and Oral and Maxillofacial Surgery University of Tripoli, School of Dentistry Tripoli Libya

**Keywords:** animal, autograft, models, sutures, swine, tissue adhesives

## Abstract

**Objectives:**

Clinician expertise influences approach choices, amidst ongoing research on suturing techniques’ biomechanical effects on graft stability. This study evaluated different suturing methods and cyanoacrylate adhesive for FGG fixation with a porcine mandible model.

**Material and Methods:**

Ninety‐four FGG procedures were performed on juvenile pig mandibles. Eight fixation methods were evaluated: seven suturing techniques—Cross Compression (CC), Vertical Circumferential Compression (VCC), Horizontal (H), CC + H, VCC + H, Miller (MIL), Holbrook and Ochsenbein (Chan et al.)—and cyanoacrylate adhesive (GLU). Outcomes measured included marginal tension (vertical displacement force) and lateral tension (horizontal displacement) at the graft margin.

**Results:**

The HOC technique demonstrated the highest marginal stability (3.82 (SD 1.35 N) and compressive resistance (0.76 (SD 0.48 N). CC + H and VCC + H also showed significantly enhanced marginal stability. Compared to MIL, HOC exhibited superior compressive resistance (*p* = 0.001), while both HOC and CC + H achieved significantly greater marginal tension (*p* < 0.05). Graft dimensions influenced outcomes: greater graft height improved compressive resistance, while increased length reduced marginal stability with horizontal sutures. Mean suturing time was 4.84 min (SD 1.55); CC was faster than MIL (*p* = 0.03), whereas HOC, CC + H, and VCC + H required significantly more time (*p* < 0.01). GLU achieved marginal stability (3.45 (SD 3.2 N) comparable to HOC.

**Conclusions:**

Within the limitations of this ex vivo study, suturing technique and graft dimensions significantly affect the FGG's biomechanical stability. Holbrook and Ochsenbein's suturing technique achieved the highest marginal stability and compressive resistance. Furthermore, tissue adhesive demonstrated comparable performance to conventional sutures, supporting its potential as an alternative fixation method.

## 1 Introduction

Free gingival grafts (FGGs) are autologous soft tissue transplants, typically harvested from the palate with the overlying epithelium, that are entirely detached and repositioned onto a prepared recipient bed within the oral cavity (Zucchelli et al. 2020). FGGs have long been a cornerstone of periodontal plastic surgery and remain the gold standard for augmenting keratinized tissue height around both natural teeth and dental implants (Horning et al. 2008; Jensen et al. 2023; Kim and Neiva 2015; Scheyer et al. 2015; Sullivan and Atkins 1968). Long‐term clinical studies report predictable keratinized tissue gains of 3.1 to 5.6 mm, with stable outcomes maintained over 20 to 30 years (Agudio et al. 2019; Agudio et al. 2016; Wennström 1996).

Graft stabilization is considered as one of the most critical determinants of FGG success (Miller 1987). Mechanically, free grafts lack intrinsic attachment strength to the recipient bed. Biologically, during the initial postoperative period, no blood vessels extend from the recipient bed into the graft, making graft survival entirely dependent on passive plasmatic diffusion through close contact with the periosteal bed (Oliver et al. 1968). Therefore, the technique used to secure and stabilize the graft is crucial to enable adequate perfusion and ensure graft viability (Oliver et al. 1968).

Several methods have been developed to achieve optimal graft stabilization, including suturing techniques (Holbrook and Ochsenbein 1983; Miller 1985), biological tissue adhesives (Hoexter 1979; Jaeger et al. 1987), and, more recently titanium tacks, and stent systems (Korkis et al. 2019; Lee et al. 2023; Zwanzig et al. 2025). Among the suturing techniques, Holbrook & Ochsenbein introduced a sophisticated technique, combining a horizontal stretching suture with vertical circumferential and crossed compressive sutures to maintain close contact between the graft and recipient bed and eliminate dead space (Holbrook and Ochsenbein 1983). In contrast, Miller advocated a more straightforward technique (MIL) that uses individual sutures at each papilla along with two apical sutures to stretch the graft and ensure adaptation to the recipient bed (Miller 1985). This approach minimizes or omits compressive sutures to reduce pressure on the graft, which has been suggested to contribute to graft contraction (Miller 1987). In practice, the choice between these two main approaches—and their intermediate variations—is largely empirical and based on clinician preference, as the biomechanical impact of different suturing techniques on graft stability has not yet been systematically investigated.

The primary aim of this ex vivo porcine model study was to compare the biomechanical performance of different FGG fixation techniques. Specifically, we evaluated graft stability and adaptation across multiple suturing methods. Additionally, we assessed the influence of graft dimensions and suture positioning, and compared conventional sutures to a cyanoacrylate‐based tissue adhesive (GLU), which has been proposed as a user‐friendly and biologically advantageous alternative (Veríssimo et al. 2021; Yilmaz et al. 2022).

## 2 Material and Methods

### 2.1 Study Design

Fresh porcine mandibles were sourced from a licensed abattoir (Michigan, USA) after routine slaughter for the food industry. No animals were sacrificed for research purposes. Per institutional policy, the University of Michigan, School of Dentistry IACUC/Ethics Committee reviewed the protocol. It deemed it exempt from animal research oversight because it involved only post‐mortem tissues. The study followed the relevant ARRIVE 2.0 items for transparent reporting of experimental research using animal materials. (Percie du Sert et al. 2020) The primary investigation focused on suturing techniques using 4‐0 silk sutures, with an additional analysis involving 5‐0 resorbable polyglycolic acid (PGA) sutures for comparative purposes.

### 2.2 Sample Collection

A total of 94 FGG procedures were performed on fresh mandibles harvested from juvenile domestic pigs (*Sus scrofa domesticus*), aged 6–8 months and weighing between 90 and 110 kg. Specimens were sourced from a licensed abattoir and stored on ice. Only jaws with intact gingiva and alveolar bone structures were included.

### 2.3 Group Allocation

Procedures were divided into two experimental arms (Table 1).

**Table 1:** Overview of the fixation techniques evaluated in the study.

Group	Technique	Key characteristics	*n* (Silk 4.0)	*n* (PGA 5.0)
**1**	CC	Diagonal cross‐compression (X‐pattern)	10	2
**2**	VCC	Vertical circumferential compression	10	2
**3**	H	Single horizontal stretching suture	10	2
**4**	CC + H	CC combined with horizontal suture	10	2
**5**	VCC + H	VCC combined with horizontal suture	10	2
**6**	MIL	Miller technique: papillary + apical periosteal sutures	10	2
**7**	HOC	Holbrook–Ochsenbein: H + CC + VCC	10	2
**8**	GLU	High‐viscosity cyanoacrylate adhesive	10	—

*Note:*Total procedures: 94 (70 silk; 14 PGA; 10 GLU).

**Figure 1:**
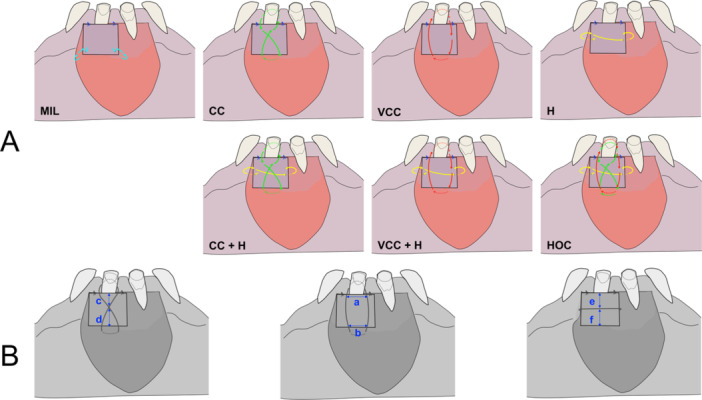
Illustrations showing (A) the different suture techniques used, including: Miller Technique (MIL)—Cross Compression (CC), Vertical Circumferential Compression (VCC), Horizontal (H), Cross Compression + Horizontal (CC + H), and Vertical Circumferential Compression + Horizontal (VCC + H); and Holbrook and Ochsenbein Technique—a horizontal stretching suture combined with vertical circumferential and crossed compressive sutures (Holbrook and Ochsenbein 1983). (B) Definition of geometric covariates for each suture configuration: horizontal distances between apical or coronal sutures (a, b); vertical distances from the suture crossing point to the graft edge (c, d); and vertical distances from the horizontal suture to the coronal and apical graft edges (e, f).

Primary Suturing Arm (Silk Sutures 4‐0 **‐** AD Surgical, Rancho Santa Margarita, CA, USA): 80 procedures, 10 per group, across 8 groups (Figure 1A):

1. Cross Compression (CC): Diagonal compressive sutures forming an X‐shape over the graft.
2. Vertical Circumferential Compression (VCC): Vertical compressive sutures placed across the graft from coronal to apical direction.
3. Horizontal (H): A single horizontal suture passed along (or close to) the graft's midline.
4. Cross Compression + Horizontal (CC + H): Combination of cross‐compression and horizontal suture.
5. Vertical Circumferential Compression + Horizontal (VCC + H): Combination of vertical compressive and horizontal suture.
6. Miller Technique (MIL): Based on the protocol proposed by Miller, this method includes interrupted sutures at the mesial and distal papillae, and two apically positioned periosteal sutures, minimizing compressive forces (Miller PD 1985).
7. Holbrook and Ochsenbein Technique: A horizontal stretching suture combined with vertical circumferential and crossed compressive sutures (Holbrook and Ochsenbein 1983).
8. Cyanoacrylate Adhesive (GLU): High‐viscosity cyanoacrylate (PeriAcryl® 90 HV‐ Glustitch Inc., Delta, BC, Canada) applied in a layered fashion beneath and over the graft, polymerized under moist gauze compression.

Additional Suturing Arm (PGA Sutures 5‐0‐ AD Surgical Premium + , Rancho Santa Margarita, CA, USA): 14 procedures (2 per group across the seven suturing configurations, excluding GLU).

Group assignments were randomized by using an online randomization tool and evenly distributed across the anterior mandibular regions.

### 2.4 Graft and Recipient Bed Preparation

All recipient bed preparation and graft harvesting were performed by a single calibrated operator (CC) using a 15 C blade. Standardized split‐thickness recipient sites were prepared on attached keratinized mucosa extending to the lining mucosa. Harvested grafts were and placed 1 mm coronal to the cementoenamel junction (CEJ). Dimensions were measured with a UNC‐15 probe:

- Graft Length: 7–10 mm
- Graft Width 5–7 mm
- Thickness: 1–2 mm

### 2.5 Suturing Technique

All suturing was performed by a single operator (KA) under 3.5× magnification. Suturing time was recorded from first needle pass to final knot. All techniques involved mesial and distal papillary stabilization, except for the GLU group.

### 2.6 Biomechanical Testing

All the biomechanical testing was done by a single investigator (KK), including two biomechanical parameters. Force was applied manually in a slow, continuous, “quasi‐static” manner using a calibrated analog force gauge, avoiding sudden or jerky movements, until the predefined endpoint was reached, consistent with commonly used comparative biomechanical protocols (Lake et al. 2023; Pastor et al. 2024).

**Figure 2:**
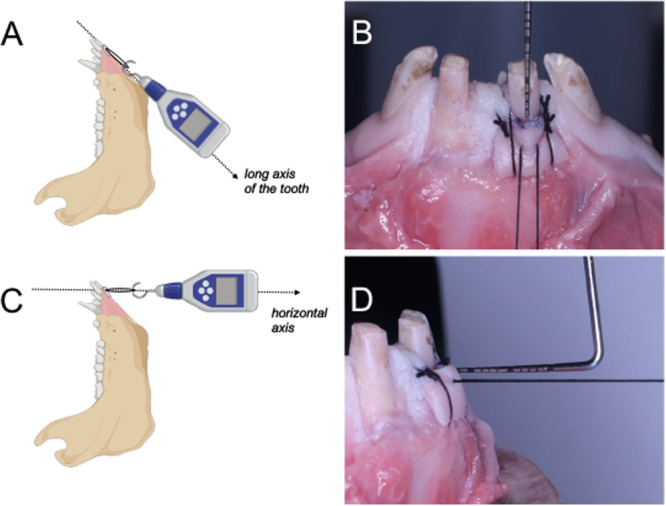
Schematic representations of the biomechanical testing setup. The parameters assessed included: marginal tension—the graft was pulled apically, parallel to the long axis of the tooth, until the cementoenamel junction (CEJ) marker became visible (A, B); and lateral compressive tension—the graft was pulled horizontally until it detached 1 mm from the root surface (C, D). All measurements were performed using a calibrated analog force gauge.

1. Marginal tension**:** The suture was passed through the central portion of the graft, approximately 2 mm apical to the coronal graft margin, and the knot was secured around the hook of the force gauge tip, creating a stable and reproducible traction point without slippage or tissue tearing. Tensile force was then applied apically, parallel to the long axis of the tooth, until the CEJ marker became visible (Figure 2A, 2B).
2. **Lateral compressive tension:** The same loop was pulled outward (horizontally) until the graft detached 1 mm from the root surface (Figure 2C, 2D).

Each measurement was conducted using a calibrated analog force gauge (Wenzhou Tripod Instrument Manufacturing Co. Ltd, Zhejiang, China) (Yoon et al. 2019; Yoon et al. 2021). Each site was tested in triplicate within a single retraction, and the average was recorded.

### 2.7 Data Collection

For each procedure, the following variables were recorded: suturing technique, suture material, graft dimensions (length, width, and thickness), and suturing time (in minutes). Suture placement distances were documented. These include:

- Vertical distances from coronal and apical graft margins,
- Horizontal distances from the graft center to both the coronal and apical edges,
- Cross‐suture distances relative to graft margins.

Biomechanical measurements included marginal tension and lateral compressive tension, with three replicates recorded per site and averaged for analysis. Due to graft detachment during marginal tension testing, lateral compressive tension was not assessed in the GLU group.

To evaluate the symmetry of suture placement, a geometric covariate was defined for each configuration (Figure 1B):

- **Cross Compression (CC):** Ratio of the longer to shorter vertical distance from the suture crossing point to the graft edge.
- **Vertical Circumferential Compression (VCC):** Ratio of the shorter to longer horizontal distance between apical or coronal sutures.
- **Horizontal (H):** Ratio of the shorter to longer vertical distance from the horizontal suture (placed at mid‐height) to the coronal and apical graft edges.

A ratio closer to 1 indicates greater geometric symmetry. This covariate was included in the statistical model to account for the potential effects of suture placement consistency on biomechanical outcomes.

### 2.8 Statistical Analysis

The initial sample size for the primary investigation using 4‐0 silk sutures was selected based on precedents in ex vivo biomechanical studies, which commonly utilize 6 to 12 specimens per condition. (Pabst et al. 2023; Pirc et al. 2025) This choice was also guided by practical constraints related to cadaver tissue availability and the intention to avoid unnecessary use of animal‐derived material. After data collection, a post‐hoc, simulation‐based sensitivity analysis was performed using the fitted mixed‐effects regression model, incorporating the observed fixed‐effect estimates and variance components via a type‐2 F‐test with Satterthwaite degrees of freedom. This analysis indicated that the design achieved power estimates exceeding 85% for detecting technique (primary predictor) related effects for both marginal (92.61%) and compressive (88.28%) tension, supporting the adequacy of the chosen sample size for the primary objective of evaluating the influence of suturing technique. In contrast, the sample size for the comparative 5‐0 PGA group was intentionally limited and was included primarily to illustrate the directional pattern in tension measurements across techniques in a qualitative manner rather than for definitive statistical comparisons. Univariate mixed‐effects linear regression models were used to evaluate each outcome variable (lateral compressive tension, marginal tension, and procedure time). To account for clustering of multiple sites within individual jaws, a random intercept for jaw was included in all models. Suture technique was treated as a fixed effect, along with graft‐related variables (length, width, and thickness) and geometric characteristics of the suture configuration, which were included as potential covariates. Model assumptions were checked through visual inspection of residuals, assessment of homoscedasticity, variance inflation factor (VIF)‐based multicollinearity diagnostics, and evaluation of influential observations. No substantial violations were detected, and sensitivity analyses confirmed the robustness of the findings.

To provide preliminary observations regarding whether the directional pattern of lateral compressive and marginal tension across suturing techniques differed between suture materials, exploratory correlation analyses were conducted separately for silk (4‐0) and PGA (5‐0) groups. Technique was treated as an ordinal numeric variable, based on its predefined factor levels, sorted by average value. Pearson correlation coefficients were computed between technique order and compressive and marginal tension within each material group. To statistically compare these correlations, a Fisher r‐to‐z transformation was applied using Steiger's method for independent correlations. This analysis was intended to explore whether the overall tension profile associated with suturing technique followed a similar trajectory across materials, rather than to estimate individual effect sizes.

All statistical analyses and data visualizations were conducted by one author (P.H.), who was blinded to group allocation, using RStudio (Version 2024.12.1 + 563, PBC) with the lme4, lmerTest, dplyr, psych, simr, and ggplot2 packages. Confidence intervals for the estimates were calculated using the Wald method, and statistical significance was set at α = 0.05 (Bates et al. 2015; Kuznetsova et al. 2017).

## 3 Results

### 3.1 Descriptive Analysis

A total of 94 FGG sites were analyzed across seven suturing techniques and one glue‐based control group (Table 2). The mean graft dimensions were as follows: length, 8.48 (SD 0.64) mm; width, 6.15 (SD 0.44) mm; thickness, 1.68 (SD 0.30) mm. The average suturing time was 4.84 min (SD 1.55). Overall, the mean marginal tension was 2.24 (SD 1.48) N, and the mean lateral compressive tension was 0.41 (SD 0.35) N.

**Table 2:** Descriptive analysis table.

Material	Technique	Number	Length (mm)	Width (mm)	Thickness (mm)	Time (minutes)	Marginal (Fenlon et al.)	Compressive (Fenlon et al.)
**Silk**	Cross compression	10	8.6 (SD 0.62	5.95 (SD 0.16	1.6 (SD 0.32	3.42 (SD 0.36	1.75 (SD 0.54	0.41 (SD 0.36)
	Cross compression + Horizontal	10	8.4 (SD 0.57	6.15 (SD 0.58	1.7 (SD 0.35	6.11 (SD 1.32	2.58 (SD 0.5	0.51 (SD 0.23)
	Horizontal	10	8.7 (SD 0.79	6.15 (SD 0.41	1.7 (SD 0.26	4.38 (SD 0.56	1.63 (SD 0.57	0.3 (SD 0.36)
	Miller	10	8.4 (SD 0.39	6.05 (SD 0.44	1.6 (SD 0.32	4.4 (SD 1.25	1.5 (SD 0.66	0.26 (SD 0.19)
	Oshsenbein	10	8.35 (SD 0.67	6.4 (SD 0.46	1.75 (SD 0.26	7.41 (SD 1.14	3.72 (SD 1.39	0.76 (SD 0.53)
	Parallel compression	10	8.65 (SD 0.82	6.1 (SD 0.62	1.65 (SD 0.34	3.75 (SD 1.03	1.66 (SD 0.58	0.24 (SD 0.25)
	Parallel + Horizontal	10	8.35 (SD 0.82	6.5 (SD 0.53	1.6 (SD 0.32	5.81 (SD 0.81	2.25 (SD 0.84	0.52 (SD 0.36)
**PGA**	Cross compression	2	8.5 (SD 0	6.5 (SD 0.71	1.75 (SD 0.35	2.64 (SD 0.13	1.4 (SD 0.57	0.28 (SD 0.08)
	Cross compression + Horizontal	2	8.5 (SD 0.71	6 (SD 0	2 (SD 0	4.56 (SD 0.18	1.83 (SD 0.04	0.40 (SD 0.06)
	Horizontal	2	9 (SD 0	6 (SD 0	2 (SD 0	3.7 (SD 0.07	1.02 (SD 0.64	0.27 (SD 0.27)
	Miller	2	8.75 (SD 0.35	6.25 (SD 0.35	1.75 (SD 0.35	2.98 (SD 0.48	1.28 (SD 0.02	0.13 (SD 0.08)
	Oshsenbein	2	8 (SD 0	6 (SD 0	1.75 (SD 0.35	5.72 (SD 0.26	4.34 (SD 1.43	0.75 (SD 0.07)
	Parallel compression	2	8 (SD 0	6 (SD 0	1.75 (SD 0.35	3.03 (SD 0.02	1.24 (SD 0.89	0.17 (SD 0.08)
	Parallel + Horizontal	2	8.5 (SD 0.71	6 (SD 0	1.5 (SD 0	5.01 (SD 0.36	1.79 (SD 0.37	0.36 (SD 0.27)
**Glue**	Glue	10	8.4 (SD 0.74	6.1 (SD 0.39	1.65 (SD 0.34	4.72 (SD 0.9	3.45 (SD 3.2	NA
**Overall**	Cross compression	12	8.58 (SD 0.56	5.96 (SD 0.14	1.62 (SD 0.31	3.29 (SD 0.44	1.69 (SD 0.54	0.39 (SD 0.33)
	Cross compression + Horizontal	12	8.42 (SD 0.56	6.12 (SD 0.53	1.75 (SD 0.34	5.85 (SD 1.34	2.46 (SD 0.54	0.49 (SD 0.21)
	Horizontal	12	8.75 (SD 0.72	6.12 (SD 0.38	1.75 (SD 0.26	4.27 (SD 0.57	1.53 (SD 0.6	0.29 (SD 0.34
	Miller	12	8.46 (SD 0.4	6.04 (SD 0.4	1.62 (SD 0.31	4.16 (SD 1.27	1.46 (SD 0.6	0.24 (SD 0.18
	Oshsenbein	12	8.29 (SD 0.62	6.33 (SD 0.44	1.75 (SD 0.26	7.12 (SD 1.23	3.82 (SD 1.35	0.76 (SD 0.48
	Parallel compression	12	8.54 (SD 0.78	6.08 (SD 0.56	1.67 (SD 0.33	3.63 (SD 0.98	1.59 (SD 0.61	0.23 (SD 0.23
	Parallel + Horizontal	12	8.38 (SD 0.77	6.42 (SD 0.52	1.58 (SD 0.29	5.68 (SD 0.81	2.18 (SD 0.78	0.49 (SD 0.34
	Glue	10	8.4 (SD 0.74	6.1 (SD 0.39	1.65 (SD 0.34	4.72 (SD 0.9	3.45 (SD 3.2	NA
**Total**	Total	94	8.48 (SD 0.64	6.15 (SD 0.44	1.68 (SD 0.3	4.84 (SD 1.55	2.24 (SD 1.48	0.41 (SD 0.35

Among the suturing techniques, the HOC method demonstrated the highest marginal (3.82 (SD 1.35 N) and lateral compressive (0.76 (SD 0.48 N) tensions. Elevated marginal tensions were also observed in the CC + H (2.46 (SD 0.54 N) and VCC + H (2.18 (SD 0.78 N) groups. The Miller technique, used as the reference, yielded marginal and compressive tensions of 1.46 (SD 0.60 N and 0.24 (SD 0.18 N, respectively. Lower compressive tensions were noted with the horizontal (0.29 (SD 0.34 N), VCC (0.23 (SD 0.23 N), and CC (0.39 (SD 0.33 N) groups.

Regression analysis suggested that suture material (silk vs. PGA) might not significantly influence marginal (*p* = 0.16) or compressive tension (*p* = 0.34), regardless of technique; however, these findings should be interpreted as preliminary observations due to the limited sample size of the 5‐0 PGA subgroup (Supplementary Table 1).

### 3.2 Suture Analysis

#### 3.2.1 Lateral Compressive Tension

According to the univariate model (Table 3), graft width was significantly associated with increased compressive tension (Estimate = 0.31; *p* < 0.001). Of all techniques, only HOC demonstrated a statistically significant improvement in compressive tension compared to Miller (Estimate = 0.49; *p* = 0.001) (Figure 4).

**Table 3:** Univariate mixed‐effects models were used to assess the influence of graft dimensions, structural configurations, and suturing techniques on lateral compressive and marginal tension.

		Univariate mixed‐effects model				
Direction	Variable	Estimate	Lower	Upper	*p* value	Samples
Compressive	Graft length	0.13	−0.01	0.26	0.059	70
	Graft width	0.31	0.14	0.47	**< 0.001**	70
	Graft thickness	0.11	−0.18	0.40	0.477	70
	Structure horizontal	0.78	−0.04	1.61	0.063	40
	Structure cross	0.30	−0.38	0.98	0.377	30
	Structure parallel	−0.53	−2.00	0.95	0.473	30
	Technique (vs. Miller)					70
	Cross compression	0.14	−0.13	0.41	0.343	
	Parallel compression	−0.03	−0.30	0.25	0.856	
	Horizontal	−0.06	−0.35	0.24	0.697	
	Cross compression + Horizontal	0.28	−0.01	0.57	0.063	
	Parallel compression + Horizontal	0.21	−0.05	0.48	0.138	
	Ochsenbein	0.49	0.21	0.76	**0.001**	
Marginal	Graft length	−0.04	−0.41	0.33	0.827	80
	Graft width	0.41	−0.10	0.91	0.119	80
	Graft thickness	0.11	−0.69	0.93	0.785	80
	Structure horizontal	−1.19	−3.62	1.25	0.330	40
	Structure cross	0.54	−1.51	2.59	0.596	30
	Structure parallel	−0.31	−4.57	3.95	0.883	30
	Technique (vs. Miller)					80
	Cross compression	0.15	−0.54	0.83	0.686	
	Parallel compression	0.10	−0.59	0.79	0.782	
	Horizontal	−0.01	−0.71	0.69	0.981	
	Cross compression + Horizontal	1.00	0.29	1.72	**0.010**	
	Parallel compression + Horizontal	0.56	−0.12	1.25	0.120	
	Ochsenbein	2.20	1.51	2.89	**< 0.001**	
	Glue	0.41	−0.30	1.12	0.277	

*Note:* Estimates, confidence intervals, and *p*‐values are reported for each variable. Bold font indicates statistical significance (*p* < 0.05)

#### 3.2.2 Marginal Tension

**Figure 3:**
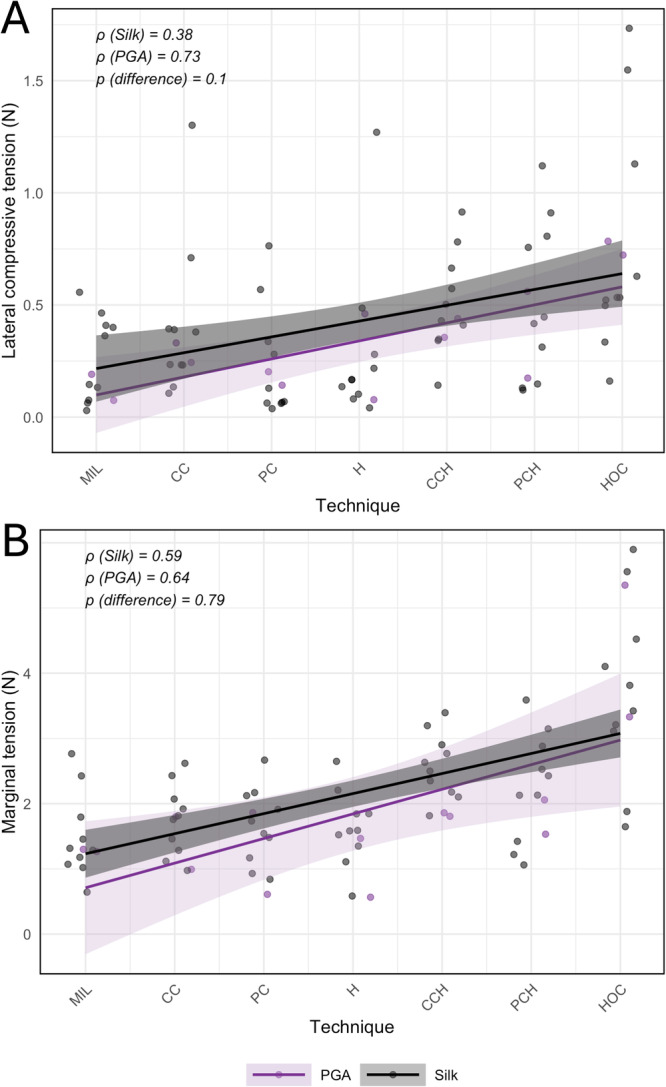
Comparison of lateral compressive tension (A) and marginal tension (B) across Suturing techniques using silk and PGA sutures. No significant differences were observed between suture materials for either parameter (*p* > 0.05).

For marginal tension, both CC + H (Estimate = 1.00; *p* = 0.010) and HOC (Estimate = 2.20; *p* < 0.001) were significantly superior to the Miller technique (Figure 3).

**Figure 4:**
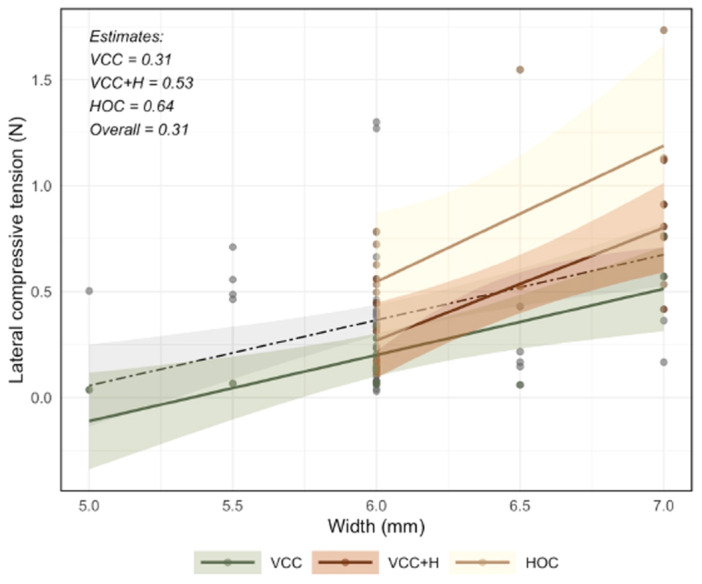
Association between graft width and lateral compressive tension for three suturing techniques: Vertical Circumferential Compression (VCC); Vertical Circumferential Compression + Horizontal (VCC + H); Holbrook and Ochsenbein Technique. Increased graft width was associated with higher tension values, particularly in HOC and VCC + H configurations. Estimates: VCC = 0.31, VCC + H = 0.53, HOC = 0.64, Overall = 0.31.

#### 3.2.3 Technique‐Specific Associations

Technique‐specific models (Table 4) revealed that:

- Graft height was positively associated with compressive tension in the VCC (*p* = 0.011), VCC + H (*p* = 0.002), and HOC (*p* = 0.048) groups.
- In the horizontal group, graft length was inversely associated with marginal tension (*p* = 0.034).
- In the HOC group, graft length was positively associated with compressive tension (*p* = 0.026).
- Additionally, in the HOC group, sutures placed closer to the graft's horizontal midline were associated with increased lateral compressive tension (*p* = 0.048).

**Table 4:** Univariate fixed‐effects models evaluating the influence of graft dimensions and structural configurations on compressive and marginal tension within each suturing technique.

			Univariate fixed‐effects model				
Technique	Direction	Variable	Estimate	Lower	Upper	*p* value	Samples
Miller	Compressive	Graft length	0.06	−0.34	0.45	0.750	10
		Graft width	−0.12	−0.47	0.22	0.451	10
		Graft thickness	0.01	−0.49	0.49	0.997	10
	Marginal	Graft length	−0.10	−1.45	1.25	0.865	10
		Graft width	−0.26	−1.46	0.94	0.630	10
		Graft thickness	0.16	−1.52	1.84	0.832	10
Cross compression	Compressive	Graft length	−0.12	−0.58	0.34	0.560	10
		Graft width	−0.66	−2.42	1.09	0.409	10
		Graft thickness	−0.02	−0.94	0.90	0.961	10
		Structure cross	−0.02	−1.37	1.33	0.974	10
	Marginal	Graft length	−0.33	−1.00	0.32	0.276	10
		Graft width	1.02	−1.64	3.69	0.402	10
		Graft thickness	−0.52	−1.85	0.81	0.393	10
		Structure cross	0.95	−0.92	2.83	0.268	10
Parallel compression	Compressive	Graft length	−0.01	−0.26	0.23	0.920	10
		Graft width	0.31	0.09	0.52	**0.011**	10
		Graft thickness	0.25	−0.32	0.82	0.342	10
		Structure parallel	−0.45	−2.83	1.92	0.671	10
	Marginal	Graft length	0.42	−0.04	0.89	0.068	10
		Graft width	0.44	−0.24	1.12	0.176	10
		Graft thickness	−0.13	−1.53	1.27	0.835	10
		Structure parallel	0.58	−4.96	6.13	0.814	10
Horizontal	Compressive	Graft length	0.20	−0.13	0.54	0.199	10
		Graft width	−0.21	−0.91	0.48	0.501	10
		Graft thickness	−0.29	−1.41	0.83	0.569	10
		Structure horizontal	−0.62	−2.85	1.59	0.534	10
	Marginal	Graft length	−0.48	−0.92	−0.04	**0.034**	10
		Graft width	−0.19	−1.31	0.92	0.702	10
		Graft thickness	−0.59	−2.33	1.13	0.449	10
		Structure horizontal	−2.44	−5.40	0.50	0.092	10
Cross compression + Horizontal	Compressive	Graft length	0.24	−0.01	0.50	0.057	10
		Graft width	0.14	−0.15	0.44	0.302	10
		Graft thickness	0.20	−0.30	0.70	0.387	10
		Structure horizontal	0.02	−1.27	1.32	0.967	10
		Structure cross	0.30	−0.37	0.97	0.329	10
	Marginal	Graft length	0.32	−0.34	0.98	0.299	10
		Graft width	−0.53	−1.08	0.01	0.055	10
		Graft thickness	0.01	−1.15	1.16	0.996	10
		Structure horizontal	−1.20	−2.44	0.03	0.058	10
		Structure cross	−0.25	−1.78	1.27	0.712	10
Parallel compression + Horizontal	Compressive	Graft length	0.17	−0.16	0.50	0.277	10
		Graft width	0.57	0.27	0.87	**0.002**	10
		Graft thickness	−0.01	−0.94	0.91	0.975	10
		Structure horizontal	1.14	−0.50	2.78	0.148	10
		Structure parallel	0.33	−2.98	3.64	0.825	10
	Marginal	Graft length	−0.62	−1.28	0.04	0.062	10
		Graft width	−0.07	−1.37	1.22	0.899	10
		Graft thickness	0.08	−2.07	2.24	0.930	10
		Structure horizontal	−1.84	−4.45	0.77	0.060	10
		Structure parallel	2.72	−4.69	10.13	0.422	10
Ochsenbein	Compressive	Graft length	0.54	0.08	1.01	**0.026**	10
		Graft width	0.73	0.01	1.45	**0.048**	10
		Graft thickness	0.08	−1.54	1.71	0.908	10
		Structure horizontal	1.87	0.02	3.71	**0.048**	10
		Structure cross	0.38	−1.88	2.64	0.710	10
		Structure parallel	0.32	−2.59	3.24	0.805	10
	Marginal	Graft length	0.68	−0.92	2.27	0.358	10
		Graft width	0.50	−1.93	2.93	0.646	10
		Graft thickness	0.58	−3.69	4.84	0.763	10
		Structure horizontal	0.51	−5.78	6.81	0.855	10
		Structure cross	−0.16	−6.18	5.86	0.953	10
		Structure parallel	3.34	−3.89	10.56	0.318	10
Glue	Marginal	Graft length	0.70	−0.72	2.12	0.290	10
		Graft width	1.06	−1.66	3.79	0.395	10
		Graft thickness	−0.34	−3.68	3.00	0.820	10

*Note:*Estimates, 95% confidence intervals, and *p*‐values are presented separately for all configurations. Bold font indicates statistical significance (*p* < 0.05)

**Figure 5:**
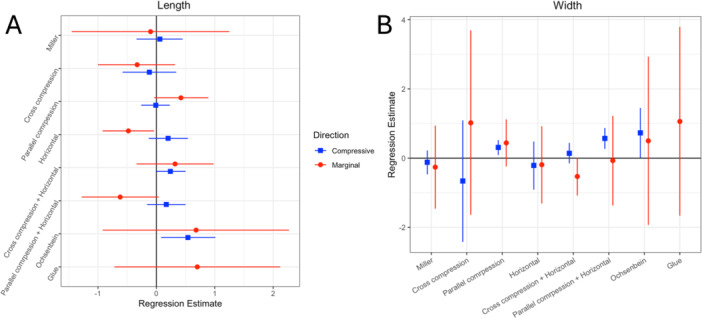
Forest plots of regression estimates showing the association of graft length (A) and graft width (B) with lateral compressive and marginal tension.

Figure 5 demonstrates the regression estimates of the association between graft length (A) and width (B).

### 3.3 Suturing Time

Suturing time varied significantly across techniques (Supplementary Table 2). Compared to the Miller technique (4.16 (SD 1.27 min), CC was significantly faster (3.29 (SD 0.44) min; Estimate = –0.97; *p* = 0.030). Conversely, longer durations were required for HOC (7.12 (SD 1.23) min; Estimate = 3.00; *p* < 0.001), CC + H (5.85 (SD 1.34) min; Estimate = 2.58; *p* = 0.001), and VCC + H (5.68 (SD 0.81) min; Estimate = 2.29; *p* = 0.002).

## 4 Discussion

The success of FGG procedures is closely linked to the mechanical stability of the graft during the early healing phase, when survival relies primarily on passive plasmatic diffusion through intimate contact with the recipient bed (Oliver et al. 1968). To our knowledge, this is the first study to quantitatively compare the biomechanical performance of different suturing techniques and cyanoacrylate fixation for FGG stabilization.

Findings indicate that complex suture configurations—including the HOC and CC + H techniques—were associated with significantly improved graft stabilization, requiring 50–100% more force for displacement than simple interrupted sutures. As expected, complex suture configurations were associated with increased procedural time. HOC, in particular, required nearly three times longer than the Miller technique, underscoring the clinical trade‐off between enhanced graft stability and surgical efficiency. Notably, the use of cyanoacrylate adhesive achieved marginal stability (3.45 (SD 3.20 N) comparable to that of the HOC suture (3.82 (SD 1.35 N), suggesting that GLU may offer a clinically viable alternative to sutures in appropriate cases.

Although silk sutures are less commonly recommended in contemporary clinical practice due to their greater propensity for bacterial accumulation and tissue reaction, they were selected for the primary experimental arm because of their favorable handling characteristics and reliable knot security. These properties facilitate the reproducible execution of complex suture configurations in controlled experimental models. The inclusion of 5‐0 PGA sutures was based on a recent review from our group, which identified 5–0 as the most commonly used diameter for FGG fixation (Negre et al. 2025). PGA sutures are extensively characterized in oral surgery, and several studies have contrasted their biological behavior with that of silk, including bacterial adhesion, inflammatory response, and histological features (Racey et al. 1978; Balamurugan et al. 2012; T R et al. 2021). Clinically, PGA demonstrates a median in‐situ survival of approximately 15 days, with tensile strength maintained for 5–7 days—sufficient to support graft stabilization during early healing (Moser et al. 1974; Shaw et al. 1996). As a non‐resorbable material, silk remains in place considerably longer but is associated with increased bacterial accumulation and higher incidence of local tissue reactions (Sortino et al. 2008; T R et al. 2021; Faris et al. 2022). These material‐specific characteristics should be considered in clinical decision‐making even when biomechanical performance appears similar.

Graft dimensions influenced mechanical stability in a technique‐dependent manner. Increased graft height enhanced compressive resistance when VCC, VCC + H, or HOC sutures were used. Conversely, greater graft length negatively affected marginal stability in the horizontal suture group. Although horizontal sutures are primarily described for graft stretching and are not used in isolation in clinical practice, their inclusion in this study was intended to provide a comprehensive biomechanical assessment.

While the role of suturing in flap and graft stabilization is well established, few studies have provided direct biomechanical comparisons of suturing techniques in periodontal plastic surgery. Shammas et al. (2020) reported no significant difference in graft contraction when stretching sutures were used in FGG procedures. At 6 months, graft height reduction was similar in both the test (33.7%) and control (33.2%) groups (Shammas et al. 2020). In a cadaver study, Tavelli et al. (2019) found that sling‐based sutures provided greater marginal stability than interrupted sutures in coronally advanced flap procedures. Recorded marginal tensions ranged from 2.83 (SD 1.02 N to 5.33 (SD 1.02) N (Tavelli et al. 2019). More recently, Pabst et al. (2023) demonstrated superior tensile strength with cyanoacrylate adhesives (GLU: 5.20 (SD 1.47 N); GLU+suture: 8.50 (SD 2.30 N) compared to sutures alone (0.88 (SD 0.61 N) in a porcine CAF model (Pabst et al. 2023). However, comparisons across studies must be interpreted with caution due to differences in suture materials, graft design, models used, and testing protocols.

Although comparison of suture material was not a primary objective of this study, clinical decision‐making should also consider material‐specific biological and handling properties. Polyglactin 910 and monofilament sutures have been associated with superior root coverage outcomes in coronally advanced flap procedures compared to silk or expanded PTFE, particularly when 5‐0 sutures were used (Ariceta et al. 2025). These findings likely reflect differences in plaque accumulation, tissue trauma, and wound healing. Timing of suture removal may also influence outcomes; early removal ( < 10 days) may impair healing, while extending beyond 14 days does not appear to provide additional benefit (Blasi et al. 2025; Tatakis and Chambrone 2016). While these data are derived from coronally advanced flap procedures, careful extrapolation to FGG is clinically reasonable.

Among alternative fixation methods, tissue adhesives offer several advantages over traditional sutures, including reduced tissue trauma, improved hemostasis, antimicrobial effects, and enhanced patient comfort (Veríssimo et al. 2021; Yilmaz et al. 2022). The mechanical performance of cyanoacrylate observed in this study suggests that adhesive fixation may provide measurable initial stability under specific conditions. This may be attributed to the uniform bonding across the graft surface achieved during polymerization, as opposed to the localized fixation provided by sutures. In addition, adhesive interaction with adjacent tooth surfaces may further enhance retention (Pabst et al. 2023). However, it is important to note that in the GLU group, graft detachment consistently occurred during primary marginal tension testing, suggesting a potential inherent weakness of adhesives under tensile stress compared with sutures. This limitation should be carefully considered when extrapolating the clinical stability of adhesive fixation relative to more complex suturing techniques. Due to the application of cyanoacrylate adhesive in two sequential layers, with a necessary setting interval between applications, the overall procedure time was not significantly shorter than that of suturing—likely attributable to the small size of the grafts. However, for larger grafts, the use of adhesive may offer a meaningful reduction in operative time.

The porcine mandibular model offers substantial translational value, as its masticatory mucosa closely approximates human tissue in epithelial thickness, keratinization, collagen architecture, and mechanical behavior (Steigmann et al. 2022; Pabst et al. 2023). However, ex vivo conditions inherently lack vascular perfusion, hemostasis, and fibrin clot formation—biological events that play a decisive role in early graft retention. In particular, fibrin clot adhesion, which contributes significantly to initial graft stabilization (Weisel and Litvinov 2017; Tutwiler et al. 2021), cannot be reproduced in a cadaveric model, thereby limiting direct extrapolation to clinical healing dynamics. These constraints are intrinsic to preclinical biomechanical models and should be considered when interpreting the findings.

Several methodological considerations also warrant acknowledgment. A specific displacement rate (mm/min) was not recorded, which may influence absolute force values due to the viscoelastic nature of soft tissues; however, identical testing conditions were applied across all groups by the same operator in a randomized order, preserving the validity of relative comparisons. Adhesive layer thickness and uniformity were not independently quantified and could have influenced fixation strength. In the cyanoacrylate group, graft detachment during marginal loading precluded lateral compressive testing; therefore, single measurements were obtained for each specimen (n = 10). As a consequence, adhesive‐related data were used solely for descriptive purposes and were not incorporated into the inferential statistical model. Importantly, all samples underwent an identical testing sequence—marginal loading followed by lateral compression when feasible—thereby minimizing the risk of systematic bias.

While mechanical stability is crucial for graft integration, the direct correlation between biomechanical parameters and clinical outcomes—such as graft survival, contraction, or esthetic outcomes—remains to be established. Importantly, this ex vivo study compares the biomechanical performance of fixation techniques; however, what constitutes a ‘clinically relevant’ force threshold is currently unknown. Therefore, it cannot be inferred whether, in specific clinical situations, the higher stability observed with the HOC technique is strictly necessary for biological healing, or whether the lower resistance achieved with the Miller technique may be sufficient. Despite these limitations, this study provides novel and clinically relevant biomechanical data on FGG fixation methods. These findings establish a foundational reference to inform surgical decision‐making and support future translational and clinical investigations.

## 5 Conclusion

Within the limitations of this ex vivo study, both suturing technique and graft dimensions were found to significantly influence the biomechanical stability of free gingival grafts. Tissue adhesive demonstrated comparable performance to conventional sutures, supporting its potential as an alternative fixation method. These findings may inform clinical decisions for optimizing graft stability.

## Author Contributions

**Kevimy Agossa:** conceptualization, writing – original draft preparation. **Khushboo Kalani, Parham Hazrati,** and **Chi‐Fan Chen:** methodology, investigation, data collection. **Parham Hazrati:** formal analysis, data curation. **Khushboo Kalani** and **Parham Hazrati:** writing – review and editing. **Chen‐Lin Tsai, Dumitru Chele,** and **Abdusalam E. Alrmali:** investigation, writing – review and editing. **Hom‐Lay Wang:** supervision, validation, writing – review and editing.

## Funding

The authors have nothing to report.

## Ethics Statement

This ex vivo study used porcine mandibles obtained post‐mortem from a licensed abattoir (juvenile pigs, 6–8 months, 90–110 kg). No animals were explicitly sacrificed for research. In accordance with institutional policy, the University of Michigan Institutional Animal Care and Use Committee (IACUC) deemed the study exempt from animal research oversight because it involved only post‐mortem tissues. The study adheres to ARRIVE 2.0 guidelines for transparent reporting of experimental research.

## Conflicts of Interest

The authors declare no conflicts of interest.

## Supporting information


**Supplementary Figure 1:** Overview of the experimental arms in the study.

**Supplementary Table 1:** Univariate Mixed Effect regression analysis regarding suture material. **Supplementary Table 2:** Univariate mixed‐effects models evaluating the effect of graft dimensions, structural configurations, and suturing technique on time.

## Data Availability

De‐identified raw data, analysis scripts, and supporting files are available from the corresponding author upon reasonable request.

## References

[cre270345-bib-0001] Agudio, G. , L. Chambrone , F. Selvaggi , and G. P. Pini‐Prato . 2019. “Effect of Gingival Augmentation Procedure (Free Gingival Graft) on Reducing the Risk of Non‐Carious Cervical Lesions: A 25‐ to 30‐year Follow‐Up Study.” Journal of Periodontology 90, no. 11: 1235–1243. 10.1002/jper.19-0032. 31194255

[cre270345-bib-0002] Agudio, G. , P. Cortellini , J. Buti , and G. Pini Prato . 2016. “Periodontal Conditions of Sites Treated With Gingival Augmentation Surgery Compared With Untreated Contralateral Homologous Sites: An 18‐ to 35‐Year Long‐Term Study.” Journal of Periodontology 87, no. 12: 1371–1378. 10.1902/jop.2016.160284. 27523520

[cre270345-bib-0003] Ariceta, A. , L. Chambrone , S. Stuhr , and E. Couso‐Queiruga . 2025. “Effect of Suturing in Root Coverage via Coronally Advanced Flaps: A Systematic Review.” Clinical Advances in Periodontics 15, no. 2: 179–190. 10.1002/cap.10312. 39276125 PMC12266327

[cre270345-bib-0004] Balamurugan, R. , M. Mohamed , H. K. R. Katikaneni , and K. A. Kumar . 2012. “Clinical and Histological Comparison of Polyglycolic Acid Suture With Black Silk Suture After Minor Oral Surgical Procedure.” The journal of contemporary dental practice 13, no. 4: 521–527. 10.5005/jp-journals-10024-1179. 23151703

[cre270345-bib-0005] Bates, D. , M. Mächler , B. Bolker , and S. Walker . 2015. “Fitting Linear Mixed‐Effects Models Using Lme4.” Journal of Statistical Software 67, no. Irct2014050717587N: 1–48. 10.18637/jss.v067.i01.

[cre270345-bib-0006] Blasi, G. , L. Maury , A. Lapedra , J. Vilarrasa , A. Monje , and J. Nart . 2025. “Suture Removal Timing and Its Effect on Root Coverage: A Randomized Clinical Trial.” International Journal of Periodontics & Restorative Dentistry, ahead of print, March 5. 1–26. 10.11607/prd.7525. 40036297

[cre270345-bib-0007] Faris, A. , L. Khalid , M. Hashim , et al. 2022. “Characteristics of Suture Materials Used in Oral Surgery: Systematic Review.” International Dental Journal 72, no. 3: 278–287. 10.1016/j.identj.2022.02.005. 35305815 PMC9275112

[cre270345-bib-0009] Hoexter, D. L. 1979. “The Sutureless Free Gingival Graft.” Journal of Periodontology 50, no. 2: 75–78. 10.1902/jop.1979.50.2.75. 370360

[cre270345-bib-0010] Holbrook, T. , and C. Ochsenbein . 1983. “Complete Coverage of the Denuded Root Surface With a One‐Stage Gingival Graft.” International journal of periodontics & restorative dentistry 3, no. 3: 8–27. 6358084

[cre270345-bib-0011] Horning, G. M. , A. Vernino , H. J. Towle, 3rd , and L. Baccaglini . 2008. “Gingival Grafting in Periodontal Practice: Results of 103 Consecutive Surgeries in 82 Patients.” International Journal of Periodontics & Restorative Dentistry 28, no. 4: 327–335. 18717371

[cre270345-bib-0012] Jaeger, U. , C. Andreoni , F. R. Kopp , and J. R. Strub . 1987. “Sutures Vs. Adhesives: Two Fixation Methods for Free Gingival Grafts. A Six‐Year Follow‐Up Study.” Quintessence international (Berlin, Germany: 1985) 18, no. 10: 691–697. 3313496

[cre270345-bib-0013] Jensen, S. S. , T. Aghaloo , R. E. Jung , et al. 2023. “Group 1 Iti Consensus Report: The Role of Bone Dimensions and Soft Tissue Augmentation Procedures on the Stability of Clinical, Radiographic, and Patient‐Reported Outcomes of Implant Treatment.” Clinical Oral Implants Research 34, no. Suppl 26: 43–49. 10.1111/clr.14154. 37750519

[cre270345-bib-0014] Kim, D. M. , and R. Neiva . 2015. “Periodontal Soft Tissue Non‐Root Coverage Procedures: A Systematic Review From the Aap Regeneration Workshop.” supplement, Journal of Periodontology 86, no. 2 Suppl: 56–72. 10.1902/jop.2015.130684. 25644300

[cre270345-bib-0015] Korkis, S. , T. N. Thompson , M. A. Vizirakis , et al. 2019. “Stabilization Techniques for Soft Tissue Grafting Around Dental Implants: Case Report.” Clinical Advances in Periodontics 9, no. 4: 192–195. 10.1002/cap.10071. 31497932

[cre270345-bib-0016] Kuznetsova, A. , P. B. Brockhoff , and R. H. B. Christensen . 2017. “Lmertest Package: Tests in Linear Mixed Effects Models.” Journal of Statistical Software 82, no. 13: 1–26. 10.18637/jss.v082.i13.

[cre270345-bib-0017] Lake, S. P. , J. G. Snedeker , V. M. Wang , H. Awad , H. R. C. Screen , and S. Thomopoulos . 2023. “Guidelines for Ex Vivo Mechanical Testing of Tendon.” Journal of Orthopaedic Research 41, no. 10: 2105–2113. 10.1002/jor.25647. 37312619 PMC10528429

[cre270345-bib-0018] Lee, W. P. , J. S. You , and J. S. Oh . 2023. “Technical Note on Simplified Free Gingival Graft Using Tack Fixation (Sfgg).” Medicina (Kaunas) 59, no. 12: 2062. 10.3390/medicina59122062. 38138164 PMC10745066

[cre270345-bib-0019] Miller, Jr., P. D. 1987. “Root Coverage With the Free Gingival Graft. Factors Associated With Incomplete Coverage.” Journal of Periodontology 58, no. 10: 674–681. 10.1902/jop.1987.58.10.674. 3478464

[cre270345-bib-0020] Miller, Jr., P. D. 1985. “Root Coverage Using the Free Soft Tissue Autograft Following Citric Acid Application. Iii. A Successful and Predictable Procedure in Areas of Deep‐Wide Recession.” International journal of periodontics & restorative dentistry 5, no. 2: 14–37. 3858263

[cre270345-bib-0021] Moser, J. B. , E. P. Lautenschlager , and B. J. Horbal . 1974. “Mechanical Properties of Polyglycolic Acid Sutures in Oral Surgery.” Journal of Dental Research 53, no. 4: 804–808. 10.1177/00220345740530040601. 4526373

[cre270345-bib-0022] Negre, A. , K. Sy , H. Sabri , K. Kalani , H. L. Wang , and K. Agossa . 2025. “Cyanoacrylate Adhesive Versus Sutures for Free Gingival Graft Fixation: A Systematic Review and Meta‐Analysis of Clinical Outcomes.” Journal of Esthetic and Restorative Dentistry 37, no. 11: 2368–2378. 10.1111/jerd.13504. 40542661 PMC12538201

[cre270345-bib-0023] Oliver, R. C. , H. Löe , and T. Karring . 1968. “Microscopic Evaluation of the Healing and Revascularization of Free Gingival Grafts.” Journal of Periodontal Research 3, no. 2: 84–95. 10.1111/j.1600-0765.1968.tb01908.x. 4249992

[cre270345-bib-0024] Pabst, A. , P. Becker , R. Kuchen , S. Schumann , and A. Kasaj . 2023. “A Comparative Study of Cyanoacrylate‐Based Tissue Adhesive and Surgical Sutures on Marginal Flap Stability Following Coronally Advanced Flap.” Clinical Oral Investigations 28, no. Irct2014050717587N: 5. 10.1007/s00784-023-05390-8. 38123821 PMC10733215

[cre270345-bib-0025] Pastor, T. , I. Zderic , K. P. van Knegsel , et al. 2024. “How Many Knots Are Necessary to Achieve Knot Security of Two High Strength Suture Tapes? A Biomechanical Comparative Analysis.” Archives of Orthopaedic and Trauma Surgery 145, no. Irct2014050717587N: 43. 10.1007/s00402-024-05638-2. 39680173

[cre270345-bib-0026] Percie du Sert, N. , V. Hurst , A. Ahluwalia , et al. 2020. “The Arrive Guidelines 2.0: Updated Guidelines for Reporting Animal Research.” PLoS Biology 18, no. 7: e3000410. 10.1371/journal.pbio.3000410. 32663219 PMC7360023

[cre270345-bib-0027] Pirc, M. , D. S. Thoma , L. Mancini , R. E. Jung , and F. J. Strauss . 2025. “Substance‐Gain Extrusion Technique (Sget) in Combination With Bopt‐Technique Description.” Journal of Esthetic and Restorative Dentistry 37, no. 9: 2060–2065. 10.1111/jerd.13494. 40390389 PMC13062715

[cre270345-bib-0029] Racey, G. L. , W. R. Wallace , C. J. Cavalaris , and J. V. Marguard . 1978. “Comparison of a Polyglycolic‐Polylactic Acid Suture to Black Silk and Plain Catgut in Human Oral Tissues.” Journal of oral surgery (American Dental Association: 1965) 36, no. 10: 766–770. PMID: 280644. 280644

[cre270345-bib-0030] Scheyer, E. T. , M. Sanz , S. Dibart , et al. 2015. “Periodontal Soft Tissue Non‐Root Coverage Procedures: A Consensus Report From the Aap Regeneration Workshop.” Journal of Periodontology 86, no. 2 Suppl: 73–76. 10.1902/jop.2015.140377. 25644301

[cre270345-bib-0031] Shammas, A. , H. Ranjbar , M. Solghar , N. Asghari , and M. Mohammadi . 2020. “Horizontal Continuous and Apical Stretching Sutures Does Not Reduce Fgg Shrinkage: A Split‐Mouth Randomized Controlled Clinical Trial.” European Oral Research 54, no. Irct2014050717587N: 42–47. 10.26650/eor.20200080. 32518910 PMC7252528

[cre270345-bib-0032] Shaw, R. J. , T. W. Negus , and T. K. Mellor . 1996. “A Prospective Clinical Evaluation of the Longevity of Resorbable Sutures in Oral Mucosa.” British Journal of Oral and Maxillofacial Surgery 34, no. 3: 252–254. 10.1016/s0266-4356(96)90280-6. 8818261

[cre270345-bib-0033] Sortino, F. , C. Lombardo , and A. Sciacca . 2008. “Silk and Polyglycolic Acid in Oral Surgery: A Comparative Study.” Oral Surgery, Oral Medicine, Oral Pathology, Oral Radiology, and Endodontology 105, no. 3: e15–e18. 10.1016/j.tripleo.2007.09.019. 18280940

[cre270345-bib-0034] Steigmann, L. , M. Steigmann , R. Di Gianfilippo , I. C. Wang , H. L. Wang , and H. L. Chan . 2022. “Comparative Assessment of Flap‐Advancing Techniques in An Ex Vivo Cadaverous Porcine Model.” International Journal of Oral & Maxillofacial Implants 37, no. 4: 823–829. 10.11607/jomi.9382. 35904840

[cre270345-bib-0035] Sullivan, H. C. , and J. H. Atkins . 1968. “Free Autogenous Gingival Grafts. I. Principles of Successful Grafting.” Periodontics 6, no. 3: 121–129. 5240496

[cre270345-bib-0036] T R, M. , S. Pal , A. K. K R , P. Bhat , and R. K. Raghupathy . 2021. “A Comparative Microbiological Study of Polyglycolic Acid and Silk Sutures in Oral Surgical Procedures.” Minerva dental and oral science 70, no. 6: 239–247. 10.23736/S2724-6329.21.04515-0. 34132506

[cre270345-bib-0037] Tatakis, D. N. , and L. Chambrone . 2016. “The Effect of Suturing Protocols on Coronally Advanced Flap Root‐Coverage Outcomes: A Meta‐Analysis.” Journal of Periodontology 87, no. 2: 148–155. 10.1902/jop.2015.150394. 26447751

[cre270345-bib-0038] Tavelli, L. , S. Barootchi , A. Ravidà , F. Suárez‐López Del Amo , G. Rasperini , and H. Wang . 2019. “Influence of Suturing Technique on Marginal Flap Stability Following Coronally Advanced Flap: A Cadaver Study.” Clinical Oral Investigations 23, no. 4: 1641–1651. 10.1007/s00784-018-2597-5. 30151706

[cre270345-bib-0039] Tutwiler, V. , R. I. Litvinov , A. Protopopova , et al. 2021. “Pathologically Stiff Erythrocytes Impede Contraction of Blood Clots.” Journal of Thrombosis and Haemostasis 19, no. 8: 1990–2001. 10.1111/jth.15407. 34233380 PMC10066851

[cre270345-bib-0040] Veríssimo, A. H. , A. K. C. Ribeiro , A. R. L. A. Martins , B. C. V. Gurgel , and R. D. A. U. Lins . 2021. “Comparative Analysis of the Hemostatic, Analgesic and Healing Effects of Cyanoacrylate on Free Gingival Graft Surgical Wounds in Donor and Recipient Areas: A Systematic Review.” Journal of Materials Science: Materials in Medicine 32, no. 9: 98. 10.1007/s10856-021-06573-z. 34406492 PMC8373739

[cre270345-bib-0041] Weisel, J. W. , and R. I. Litvinov . 2017. “Fibrin Formation, Structure and Properties.” In Subcellular Biochemistry 82: 405–456. 10.1007/978-3-319-49674-0_13. PMC5536120 28101869

[cre270345-bib-0042] Wennström, J. L. 1996. “Mucogingival Therapy.” Annals of Periodontology 1, no. Irct2014050717587N: 671–701. 10.1902/annals.1996.1.1.671. 9118276

[cre270345-bib-0043] Yilmaz, M. , S. Kayaalti‐Yüksek , and B. Karaduman . 2022. “The Effects of Cyanoacrylate on Clinical Healing and Self‐Reported Outcomes Following Free Gingival Graft Surgery: A Randomized Clinical Study.” Clinical and Experimental Health Sciences 12: 691–696. 10.33808/clinexphealthsci.1012775.

[cre270345-bib-0044] Yoon, C. S. , H. B. Kim , Y. K. Kim , H. Kim , and K. N. Kim . 2019. “Keystone‐Design Perforator Island Flaps for the Management of Complicated Epidermoid Cysts on the Back.” Scientific Reports 9, no. Irct2014050717587N: 14699. 10.1038/s41598-019-51289-4. 31605009 PMC6789127

[cre270345-bib-0045] Yoon, C. S. , Y. T. Kong , S. Y. Lim , J. Kim , H. W. Shin , and K. N. Kim . 2021. “A Comparative Study for Tension‐Reducing Effect of Type I and Type Ii Keystone Perforator Island Flap in the Human Back.” Scientific Reports 11, no. Irct2014050717587N: 16699. 10.1038/s41598-021-96272-0. 34404867 PMC8371087

[cre270345-bib-0046] Zucchelli, G. , L. Tavelli , M. K. McGuire , et al. 2020. “Autogenous Soft Tissue Grafting for Periodontal and Peri‐Implant Plastic Surgical Reconstruction.” Journal of Periodontology 91, no. Irct2014050717587N: 9–16. 10.1002/jper.19-0350. 31461778

[cre270345-bib-0047] Zwanzig, K. , S. Akhondi , L. Tavelli , and A. Lanis . 2025. “The Use of Titanium Pins for the Management and Fixation of Free Gingival Grafts and Apically Repositioned Flaps During Vestibuloplasty: A Technique Report.” International journal of periodontics & restorative dentistry 45, no. 3: 395–405. 10.11607/prd.7197. 38820278

