# Supplementary material for: Effect of Suturing and Adhesive Fixation on Free Gingival Graft Stability: An Ex‐Vivo Porcine‐Model Study
Source: Clin Exp Dent Res. 2026 Apr 26;12(2):e70345. doi: 10.1002/cre2.70345 (PMC13110839; doi:10.1002/cre2.70345)
Supplement: Supplementary file 2 — Supplementary Table 1: Univariate Mixed Effect regression analysis regarding suture material. Supplementary Table 2: Univariate mixed‐effects models evaluating the effect of graft dimensions, structural configurations, and suturing technique on time. [file CRE2-12-e70345-s002.docx]

Supplementary Material

|  |  | **Univariate mixed-effects model** | | | |  |
| --- | --- | --- | --- | --- | --- | --- |
| **Suture** | Direction of tension | Estimate | Lower | Upper | P value | Samples |
| **PGA (compared to silk)** | Lateral compressive | -0.09 | -0.27 | 0.09 | 0.342 | 84 |
|  | Marginal | -0.31 | -0.75 | 0.12 | 0.166 | 84 |

Supplementary Table 1- Univariate Mixed Effect regression analysis regarding suture material.

|  |  | **Univariate mixed-effects model** | | | |  |
| --- | --- | --- | --- | --- | --- | --- |
| **Direction** | Variable | Estimate | Lower | Upper | P value | Samples |
| **Time** | Graft length | -0.28 | -0.87 | 0.31 | 0.354 | 70 |
|  | Graft width | 0.76 | -0.03 | 1.54 | 0.060 | 70 |
|  | Graft thickness | 0.24 | -1.08 | 1.55 | 0.717 | 70 |
|  | Structure horizontal | 2.43 | -0.58 | 5.44 | 0.110 | 40 |
|  | Structure cross | 0.89 | -2.36 | 4.15 | 0.579 | 30 |
|  | Structure parallel | -0.37 | -3.04 | 2.30 | 0.704 | 30 |
|  | Technique (vs. Miller)  Cross compression  Parallel compression  Horizontal  Cross compression + Horizontal  Parallel compression + Horizontal  Ochsenbein | -0.97  -0.65  -0.02  1.69  1.41  3.00 | -1.85  -1.53  -0.90  0.83  0.53  2.13 | -0.10  0.23  0.86  2.58  2.29  3.89 | **0.030**  0.143  0.96  **0.001**  **0.002**  **< 0.001** | 70 |

Supplementary Table 2- Univariate mixed-effects models evaluating the effect of graft dimensions, structural configurations, and suturing technique on time. Estimates with confidence intervals and p-values are reported for each variable.

Supplementary Figure 1- Overview of the experimental arms in the study. (A) Primary Suturing Arm: silk sutures (4-0) with a representative example of the vertical circumferential compression (VCC) suture technique. (B) Additional Suturing Arm: polyglycolic acid (PGA) sutures (5-0) with a representative example of the VCC technique. (C) Cyanoacrylate Adhesive Arm (GLU): high-viscosity cyanoacrylate (PeriAcryl® 90 HV) applied in a layered fashion beneath and over the graft and polymerized under moist gauze compression.
