# Supplementary material for: Effect of Suturing and Adhesive Fixation on Free Gingival Graft Stability: An Ex‐Vivo Porcine‐Model Study
Source: Clin Exp Dent Res. 2026 Apr 26;12(2):e70345. doi: 10.1002/cre2.70345 (PMC13110839; doi:10.1002/cre2.70345)
Supplement: Supplementary file 1 — Supplementary Figure 1: Overview of the experimental arms in the study. [file CRE2-12-e70345-s001.pdf]

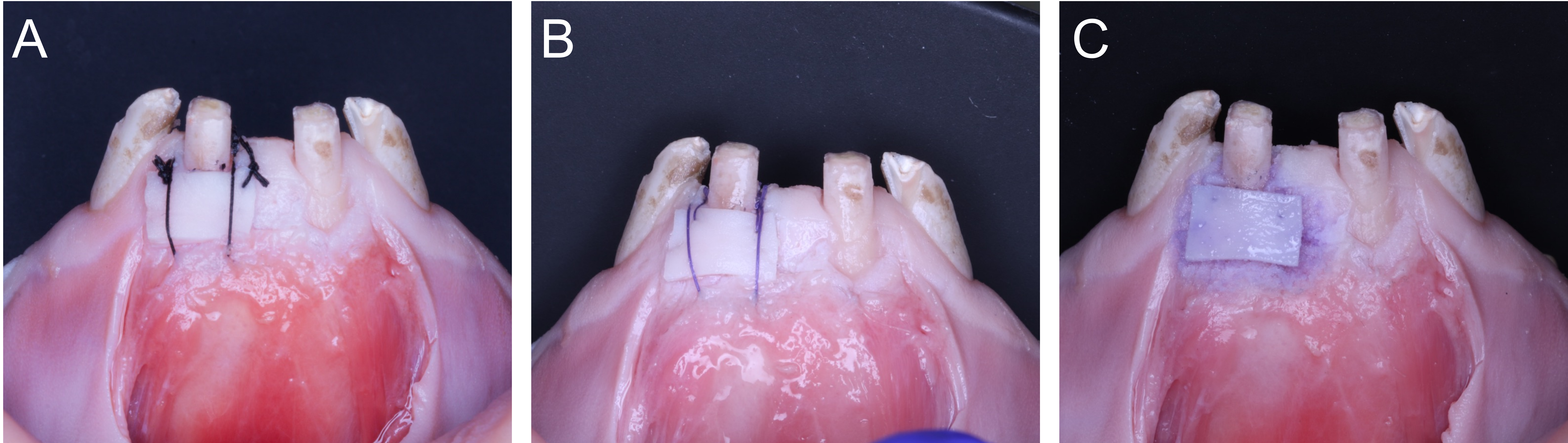
A
B
C
